# Relationship between fall history and toe grip strength in older adults with knee osteoarthritis in Japan: A cross-sectional study

**DOI:** 10.1371/journal.pone.0282944

**Published:** 2023-03-13

**Authors:** Yuya Mawarikado, Yusuke Inagaki, Tadashi Fujii, Takanari Kubo, Akira Kido, Yasuhito Tanaka

**Affiliations:** 1 Graduate School of Medicine, Musculoskeletal Reconstructive Surgery, Nara Medical University, Nara, Japan; 2 Department of Rehabilitation Medicine, Nara Medical University, Nara, Japan; 3 Department of Orthopeadic Surgery, Kashiba Asahigaoka Hospital, Nara, Japan; 4 Department of Rehabilitation, Osaka Kawasaki Rehabilitation University, Osaka, Japan; 5 Department of Orthopaedic Surgery, Nara Medical University, Nara, Japan; Baqai Medical University, PAKISTAN

## Abstract

**Background:**

Knee osteoarthritis (KOA), one of the most common musculoskeletal diseases in older adults, is associated with a high incidence of falls. Similarly, toe grip strength (TGS) is associated with a history of falls in older adults; however, the relationship between TGS and falls in older adults with KOA who are at risk of falling is not known. Therefore, this study aimed to determine if TGS is associated with a history of falls in older adults with KOA.

**Methods:**

The study participants, older adults with KOA scheduled to undergo unilateral total knee arthroplasty (TKA), were divided into two groups: non-fall (n = 256) and fall groups (n = 74). Descriptive data, fall-related assessments, modified Fall Efficacy Scale (mFES), radiographic data, pain, and physical function including TGS were evaluated. The assessment was conducted on the day before performing TKA. Mann–Whitney and chi-squared tests were performed to compare the two groups. Multiple logistic regression analysis was performed to determine the association of each outcome with the presence or absence of falls.

**Results:**

Mann-Whitney U test revealed that the fall group had statistically significantly lower height, TGS on the affected and unaffected sides, and mFES. Multiple logistic regression analysis revealed that the incidence of fall history is associated with TGS on the affected side; the weaker the affected TGS of the KOA, the more likely the individual is to fall.

**Conclusions:**

Our results indicate that TGS on the affected side is related to a history of falls in older adults with KOA. The significance of evaluating TGS among patients with KOA in routine clinical practice was demonstrated.

## Introduction

Falls in older adults can lead to injuries, such as bone fractures, and to significant deterioration of physical function. Over 30% of people aged 65 or older experience approximately one fall every year [1]. Knee osteoarthritis (KOA) is one of the most common musculoskeletal diseases in older adults. It has been reported that an increased history of falls and hip fractures is associated with an increase in knee pain, and the incidence of non-vertebral fractures is 1.6 times higher in older adults with KOA than in those without [2]. The incidence of falls in older adults with KOA is approximately 30% higher than that in healthy older adults, and approximately half of individuals aged 60 years or older fall at least once annually [3, 4]. Older adults with KOA and a fall history are more likely to fall after total knee arthroplasty (TKA) than a cohort of older adults with KOA who do not have a history of falls [5, 6].

As mentioned, older adults with KOA have a high incidence of falls; however, related risk factors remain undetermined in this population. Knee pain, impaired balance, lower muscle weakness, and decreased walking ability have been reported as risk factors for falls in individuals with KOA [7, 8]. Previous studies on older adults without KOA indicated that clinical benchmarks, such as Timed Up and Go (TUG) [9, 10], fall-related self-efficacy [11], knee extension strength [12], and toe grip strength (TGS) [13–15] are associated with the risk of a fall. These outcomes may be even stronger predictors in individuals with KOA, who generally have impaired mobility and muscle strength.

Among the aforementioned risk factors for falls, particular attention has been paid to TGS. Many studies reported an association between TGS and falls in older adults. With increasing age, TGS in older adults deteriorates [16–18], resulting in decreased walking speed and static balance ability [19–21]. TGS training to nursing home residents led to significant improvements in the fall risk index (-1.4 points). A significant increase in TGS was noted in the intervention group compared to that in the non-intervention group (Intervention group increased 1.9 kg, non-intervention group decreased 0.2 points) [22]. Therefore, it is clinically important to evaluate and strengthen TGS to prevent falls in older adults.

To date, no study has investigated the relationship between falls and TGS in older adults with KOA who are at risk of falling owing to lower limb muscle weakness and impaired balance. We hypothesized that the history and frequency of falls in older adults with KOA would be associated with TGS. Therefore, this study aimed to determine whether TGS is associated with a history of falls in older adults with KOA.

## Methods

### Ethical issues

This study complied with the Declaration of Helsinki and was approved by the Research Ethics Committee of Kashiba Asahigaoka hospital (2019-04-21-007). Details of the study protocol and aim were explained to all participants, both verbally and in writing. All study participants then signed a written consent prior to participating in the study.

### Participants

This study used a descriptive cross-sectional design to identify the association between falls and clinical evaluation factors including TGS. We recruited 407 participants with KOA, scheduled to undergo unilateral TKA at a single hospital in Japan between May 2019 and September 2021. The inclusion criteria were: 1. diagnosis of KOA, 2. ability to ambulate independently or with a T-cane at the time of pre-operative evaluation, 3. individuals who were scheduled for primary TKA, 4. individuals between 60 and 84 years of age, and 5. informed consent to participate in the study was obtained. The exclusion criteria were: 1. diagnosis of rheumatoid arthritis, idiopathic osteonecrosis, or foot and ankle disorders; 2. individuals with bilateral toe flexion problems, neurologic diseases, or other musculoskeletal diseases that significantly impair basic movements, such as walking; and 3. Those with severe depression or dementia, which would hinder evaluation. The number of falls over one-year period was obtained retrospectively within the inclusion time by nurses. The group with one or more falls was defined as the fall group, and the group with no falls was defined as the non-fall group.

### Experimental procedure

We assessed the participants one day prior to TKA. Descriptive data, fall-related assessments (presence of falls and fear of falling), radiographs, and physical function data were collected from electronic medical records. Physical function measurements were measured in the rehabilitation room, and were performed by 14 randomly assigned physical therapists for all participants to reduce bias as much as possible. The surgical side was considered as the affected side, whereas the non-surgical side was considered as the unaffected side.

### Fall definition

A fall was defined as “an event that results in a person coming to rest unintentionally on the ground or other lower level, not as a result of a major intrinsic event of overwhelming hazard” [23]. Falls were excluded if they were not related to gait, standing and transfer, for example a fall with a bicycle and ladder.

### Primary outcome

**Fig 1:**
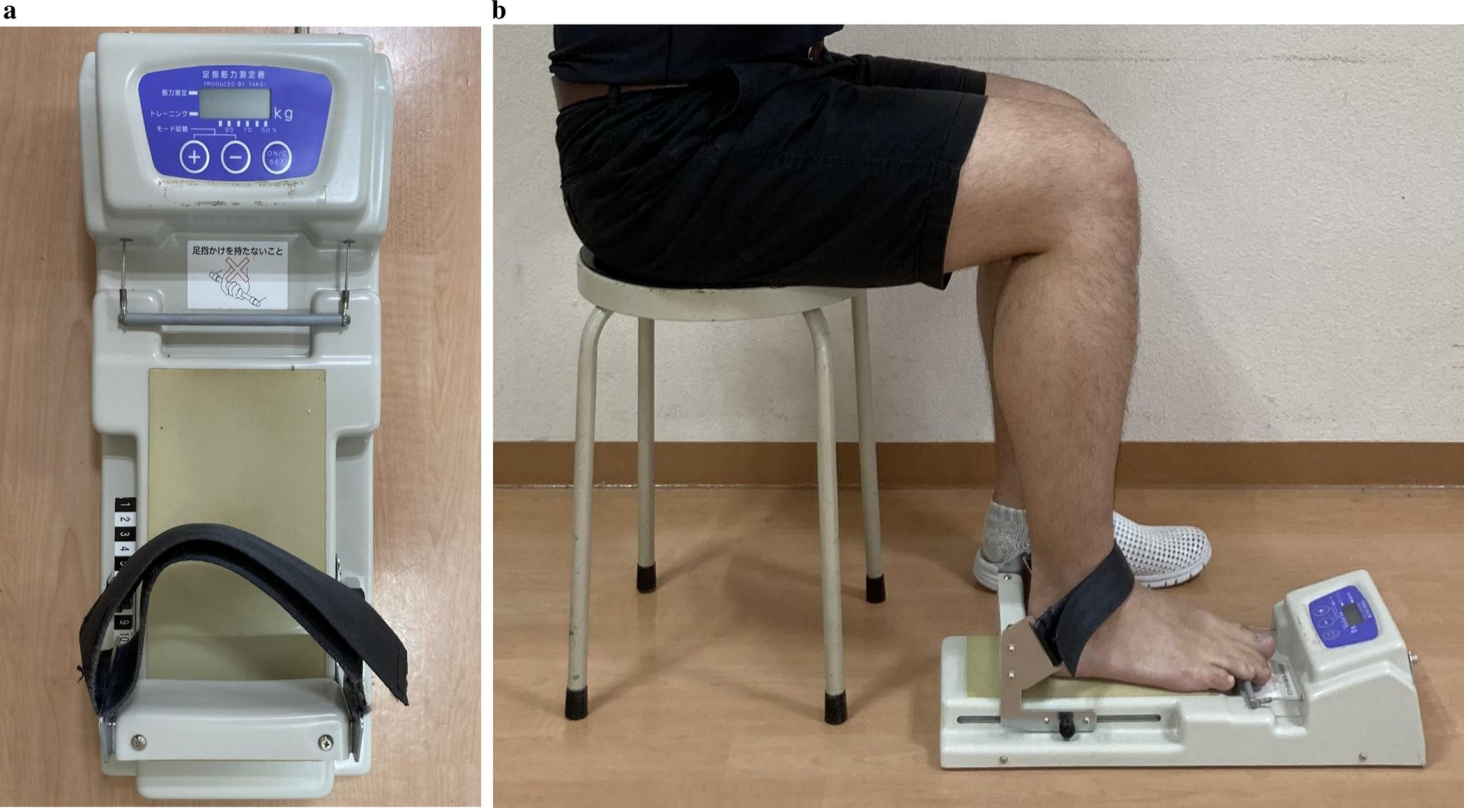
Toe grip strength assessment. a. Toe grip dynamometer employed to measure toe grip strength. b. The grip bar of the instrument was adjusted to the first metatarsophalangeal joint of the participant. The participants sat on the edge of their seats keeping their trunks in a vertical position and the hip and knee joints bent to approximately 90°.

A toe grip dynamometer (T.K.K.3362; Takei Scientific Instruments, Niigata, Japan) was used to measure TGS (Fig 1) in a sitting position with 90° hip and knee joint flexion and the ankle in a neutral position. Under vertical loading on the foot, the plantar aponeurosis was extended with the foot truss structure [24]. The participants were instructed to place their test foot within the heel stopper and to grasp the dynamometer grip bar with their toes. They first performed a few test contractions with maximum effort to familiarize themselves with the measurement process and then performed as many voluntary isometric contractions as possible. Maximum TGS was measured twice, and the mean value (kg) was calculated. Participants performed maximum-effort contractions after the "warm-up" repetitions. An almost perfect inter- and intra-rater reliability of this measurement protocol using the toe grip dynamometer has been observed previously in people aged 60–79 years [25].

### Secondary outcomes

Descriptive data, such as gender, age, height, weight, and body mass index were collected by nurses during the evaluation. The patients self-reported whether and how many times they experienced falls in the one past year. The severity of KOA was determined using the Kellgren–Lawrence (K-L) grading system [26]. Four orthopedic surgeons evaluated all medical records and determined the K-L grade severity. Isometric knee extension strength (IKES) was measured using a hand-held dynamometer (μ-tas F1, ANIMA, Tokyo, Japan) with participants in a seated position and the knee in 90° flexion [27]. The reliability and validity of this measurement method have been previously demonstrated in patients with KOA [27, 28]. The participants were instructed to increase the intensity of knee extension against the dynamometer gradually and for approximately 2 seconds to avoid explosive contraction, and to maintain their maximal force output for approximately 3 seconds. Maximum IKES was measured twice, and the mean value (kg) was calculated. Pain levels at rest and during walking were determined using a visual analog scale ranging from 0 (no pain) to 100 mm (worst pain) [29]. This measurement method has been reported to be reliable and valid for the assessment of individuals with KOA [30]. The TUG was used as a behavioral measure of knee function using standard test methods [31]. The reliability and validity of this measurement method have been demonstrated [32, 33]. The participants stood up from an armless chair upon the assessor’s signal, walked to the 3-m point, and returned to sit on the same chair. The TUG measurements were recorded twice and a mean value between the two measurements was calculated and used for statistical analysis. Fear of falling was assessed using the Japanese version of the modified Fall Efficacy Scale (mFES), developed by Hill et al [34]. The mFES is a 10-grade scale comprising 14 items (score range: 0–140 points), and a modified version of the Falls Efficacy Scale developed by Tinetti et al. [35]. The mFES is used to determine the level of confidence in performing specific movements and actions without falling, with higher scores indicating higher levels of self-efficacy in fall prevention and less fear of falls. The reliability and validity of this measurement method have been demonstrated [34].

### Data analysis

Descriptive data were presented as the number of cases, mean with standard deviations (SD), and percentages. For all analyses, the significance level was set at 5%. All statistical analyses were performed using SPSS Statistics for Windows, version 26.0 (IBM Corp, Tokyo, Japan). The sample size was calculated by G-power Post-hoc. The sample sizes for both the fall and non-fall groups were calculated based on effect size = 0.5 and α = 0.05.

Before comparing the difference between the two groups, a Kolmogorov–Smirnov test was performed as a homogeneity test. The results confirmed that the p-values for all factors were less than 0.05 for both groups. Therefore, Mann–Whitney- and chi-squared tests were used to test the difference between the two groups. The factors subjected to Mann–Whitney U test were age, height, weight, BMI, TGS on both sides, IKES on both sides, pain at rest on both sides, pain while walking on both sides, TUG, and mFES. Factors subjected to the chi-squared test were sex and K-L grade.

Multiple logistic regression was performed to examine the association of each factor to the dependent variable (i.e., fall or no fall history). The independent variables were height, weight, TGS on both sides, and mFES. Hosmer-Lemeshow test was performed to determine whether the analysis result was significantly fitted to the actual data. The significance of the regression equation was confirmed by rate of accurate discrimination. The variance inflation factor (VIF) was calculated to account for the degree of multicollinearity among the related factors.

## Results

**Fig 2:**
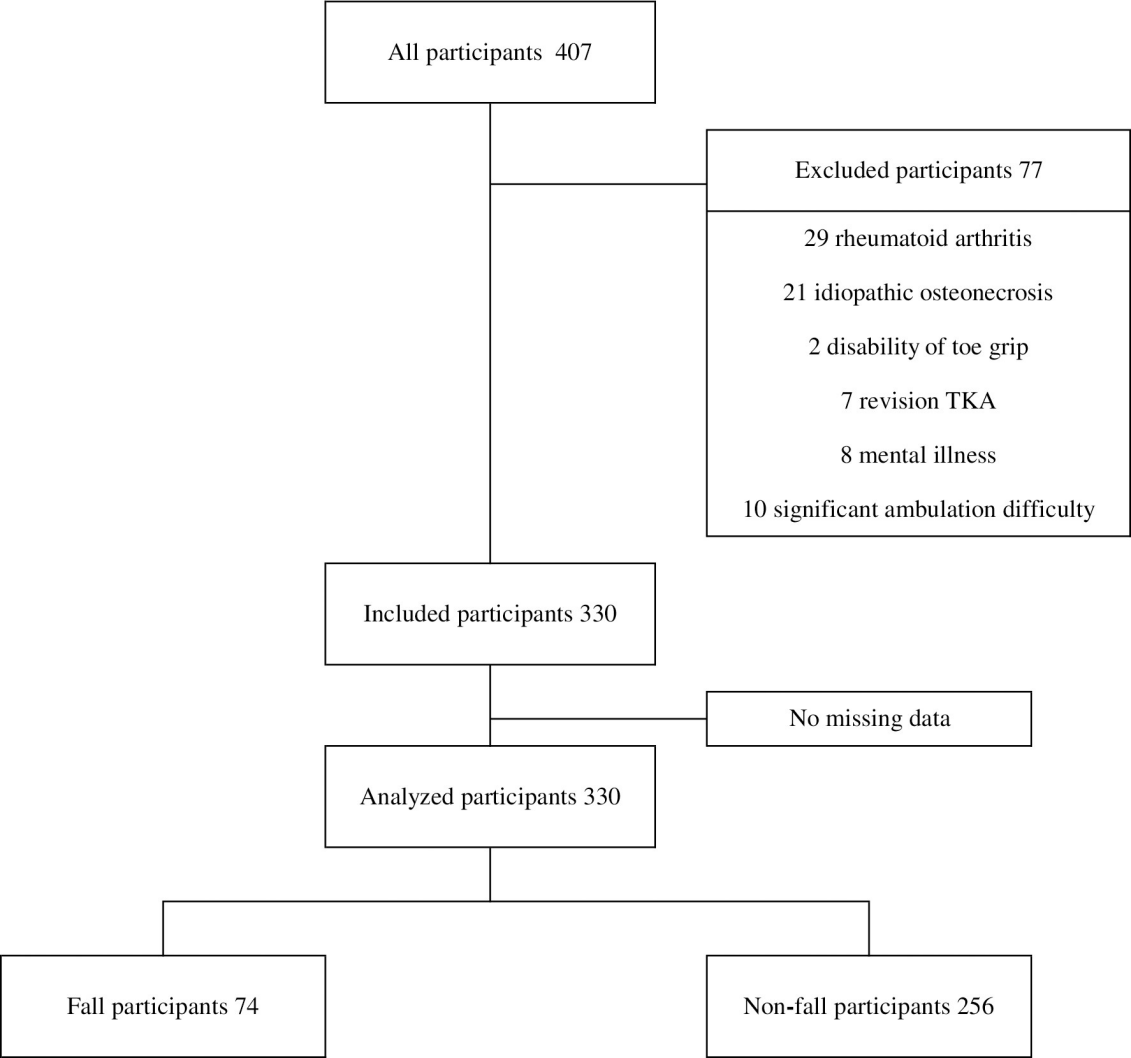
Flow diagram of the study participants’ inclusion process.

We excluded 77 participants, while 330 met the inclusion criteria (Fig 2). The 330 participants were divided into two groups: fall and non-fall. The participants’ descriptive characteristics and results of Mann–Whitney- and chi-squared tests are summarized in Table 1. Of the 330 participants enrolled in the study, 74 (22.4%) were in the fall group and 256 (77.6%) were in the non-fall group. The percentage of fall was calculated from the 330 participants who met the inclusion criteria of the study. Of the participants with a fall history, 42 reported 1 fall, 14 reported 2 falls, 10 reported 3 falls, 4 reported 4 falls, and 4 reported 5 falls. A history of 6 or more falls was never recorded. The mean number of falls per participant was 1.85. In addition, the sample size was calculated with 74 patients in the fall group and 256 in the non-fall group, the power was 0.97.

**Table 1:** Participants’ descriptive characteristics and descriptive statistics for the study variables (n = 330).

		Fall group (n = 74)			Non fall group (n = 256)			p-value	Phi coefficient
Percentage of fall, %		22.4							
Gender, (female/male)		58/16			194 / 62			0.643	-0.025
Age, years		73.7	±	8.1	74.8	±	7.2	0.396	
Height, cm		152.6	±	8.6	154.9	±	8.6	0.014*	
Weight, kg		61.5	±	12.9	63.0	±	12.0	0.247	
BMI, kg/m^2^		26.3	±	4.0	26.2	±	3.9	0.898	
K-L grade, n (%)	Ⅱ	0 (0)			5 (2.0)			0.361	0.079
	Ⅲ	6 (8.1)			28 (10.9)				
	Ⅳ	68 (91.9)			223 (87.1)				
TGS, kg	affected side	7.0	±	4.3	8.6	±	4.3	0.003*	
	unaffected side	7.1	±	4.0	8.7	±	4.5	0.007*	
IKES, kg	affected side	15.0	±	7.2	15.8	±	9.2	0.989	
	unaffected side	17.2	±	6.9	18.2	±	11.1	0.715	
Rest pain, mm	affected side	15.4	±	19.6	13.1	±	20.6	0.205	
	unaffected side	5.6	±	12.0	5.0	±	13.3	0.110	
Walking pain, mm	affected side	50.6	±	22.3	46.2	±	26.2	0.158	
	unaffected side	23.8	±	24.3	19.4	±	23.9	0.089	
TUG, s		12.6	±	4.2	12.8	±	4.5	0.989	
mFES, point		98.4	±	35.5	113.0	±	28.1	0.001*	

Data were expressed as mean (standard deviation). *P<0.05

BMI, Body mass index; K-L grade, Kellgren-Lawrence grade; TGS, Toe grip strength; IKES, Isometric knee extension strength; TUG, Timed up and go test; mFES, modified Fall Efficacy Scale

Factors that differed significantly between the fall and non-fall groups were height (p = .014), TGS on the affected side (p = .003), TGS on the unaffected side (p = .007), and mFES (p = .001).

The results of multiple logistic regression analysis are presented in Table 2. The model chi-squared test revealed significant results, indicating association with TGS on the affected side (β = -.081, p = .024, Odds ratio [OR] = .922), and mFES (β = -.013, p = .002, OR = .987). The model χ^2^ was significant at p < .01. The result of the Hosmer–Lemeshow test was not significant at p = .56, and the fit of regression equation was good. The rate of accurate discrimination was 77.6%. VIF for mFES and TGS on the affected side were 1.023 in both cases, and no multicollinearity was observed. VIF calculated from the related factors were height: 1.165; weight: 1.059; and TGS on the unaffected side: 2.320.

**Table 2:** Results of the multiple logistic regression analysis.

	B	Standard Error	Wald	p-value	Odds ratios
					(95% confidence interval)
TGS on the affected side	-0.081	0.036	5.069	0.024	0.922 (0.86–0.99)
mFES	-0.013	0.004	9.673	0.002	0.987 (0.98–0.99)
Constant	0.760	0.494	2.370	0.124	

Regression equation: ln(p/1-p) = {-.081(TGS on the affected side)}+{-.013(mFES)}+.760 model x^2^, p < .01; Hosmer-Lemeshow test, P = .56; rate of accurate discrimination, 77.6% TGS, Toe grip strength; mFES, modified Fall Efficacy Scale

## Discussion

We analyzed a number of factors that potentially contributed to a history of falls in patients with KOA, including TGS, and clarified which of those had an impact. Only a few studies reported the incidence of falls in individuals with KOA in Japan [36]. To the best of our knowledge, this is the first study to investigate the relationship between history of falls and TGS in older adults with KOA. Our results indicated that falls in older adults with KOA were related to TGS. In short, falls in older adults with KOA are associated with lower TGS.

### The percentage of fall rate in this study

In a study of 5,062 frail older adults in Japan, approximately 30% experienced a fall at least once in a year [37]. Compared to older adults with KOA in Australia (48% fell within 12 months prior to TKA) [4] and in the United Kingdom (24% fell within 3 months prior to TKA) [5], the fall rate 12 months prior to TKA in our study was lower (22.4%). However, according to the publication of vital statistics in Japan, accidental deaths from falls among older adults are on the rise. The overall mortality rates per 100000 persons in the older population increased from 19.5 in 1997 to 20.5 in 2016 [38]. A previous study has reported that fall history before performing TKA increased the risk of post-operative falls [5, 6]. This insight might lead to a better understanding of prevention of injuries from post-operative falls.

### Toe grip strength and fall history

A previous study has reported that TGS declines with age [39], resulting in diminished walking ability and static balance, which may be risk factors for falls. Tsuyuguchi et al. recruited middle-aged adults and, whose average age was 62.02, divide them into high and low risk of falls. They found TGS to be an independent risk factor for fall occurrence [40]. However, there is no report on whether reduced TGS in individuals with KOA is associated with the fall itself. Based on the results of our study, we believe that TGS contributes to the challenges faced by older adults with KOA and a fall history. Although a detailed causal relationship is unknown, multiple regression analysis has identified TGS as an independent factor associated with KOA [41]. Conversely, abnormal loading of the knee joint can be caused by changes in the kinematic relationship between the foot and knee [42, 43]. Compared to healthy older adults, those with KOA have lower TGS, and the measured pressure decreases during walking [44, 45]. It is possible that the progression of KOA leads to decreased TGS; conversely, decreased TGS may contribute to KOA progression. However, this causal relationship is unclear; therefore, further studies are required to investigate it. Regardless of the causal pathway, there is interdependence between TGS and KOA, which increases the risk of falls. In the future, studies should approach the causal relationship between KOA and TGS from the perspectives of kinesiology and biomechanics, in addition to seeking strategies to prevent falls.

### Self-efficacy for falls and fall history

Adults with KOA with more frequent falls may have a more pronounced fear of falling than those with fewer falls. Tinetti et al. [46] defined fear of falling as "anxiety about falling that causes one to avoid activities of daily living, even though one is capable of performing them”. In their study, fear of falling depended on the history of falls, ranging from 12–65% among community-dwelling older adults without a fall history, and from 29–92% among those with [47]. Therefore, fall history is associated with fear of falling [48]. Our results support those of previous studies [48]. In addition, participants with KOA and a history of falls experienced knee pain, knee instability, and muscle weakness in the lower extremities, which could have also contributed to a greater fear of falling. Post-fall syndrome, in which a loss of self-confidence after a fall leads to a decreased activity level, in turn amplifies the fear of falling.

### Limitations

This study had three major limitations. First, TGS was the only assessment performed on the foot. The degree of flatfoot and range of motion of the foot, which are common problems in individuals with KOA, were not measured. The abnormal foot posture of KOA has excessive first medial tibiofemoral contact force during walking [49]. As these factors are also associated with gait, which is involved in half of all fall scenarios [50], they are likely to contribute to falls. Second, previous studies have reported decreased physical activity [51] as well as hip [52] and ankle [53] weakness as risk factors for falls. Because we did not evaluate these factors and did not include these results in our regression analysis, we could not determine the relative contribution of TGS to physical activity and muscle strength for the above factors in older adults with KOA. Finally, we conducted a cross-sectional observational study; therefore, subsequent cohort and intervention studies should be conducted to better clarify the causal relationships between foot function and falls in older adults with KOA.

## Conclusions

In this study, we investigated the factors leading to falls in older adults with KOA in Japan. Our results indicated that falls in older adults with KOA were related to TGS. In short, falls in older adults with KOA are associated with lower TGS. Physical therapy interventions to enhance TGS could be one solution to help prevent falls in individuals with KOA. In the future, cohort and interventional studies evaluating the relationship between TGS and falls should be performed.

## Supporting information

S1 Checklist STROBE statement—checklist of items that should be included in reports of *cross-sectional studies*. (DOCX)

S1 Dataset (XLSX)
